# The promise of machine learning applications in solid organ transplantation

**DOI:** 10.1038/s41746-022-00637-2

**Published:** 2022-07-11

**Authors:** Neta Gotlieb, Amirhossein Azhie, Divya Sharma, Ashley Spann, Nan-Ji Suo, Jason Tran, Ani Orchanian-Cheff, Bo Wang, Anna Goldenberg, Michael Chassé, Heloise Cardinal, Joseph Paul Cohen, Andrea Lodi, Melanie Dieude, Mamatha Bhat

**Affiliations:** 1 Ajmera Transplant Program, University Health Network, Toronto, ON Canada; 2 Department of Medicine, University of Ottawa, Ottawa, ON Canada; 3 Department of Gastroenterology, Toronto General Hospital Research Institute, Toronto, ON Canada; 4 Division of Gastroenterology, Department of Medicine, Vanderbilt University Medical Center, Nashville, TN USA; 5 Department of Cell and Systems Biology, University of Toronto, Toronto, ON Canada; 6 Library and Information Services, University Health Network, Toronto, ON Canada; 7 Vector Institute for Artificial Intelligence, Toronto, ON Canada; 8 Department of Medicine (Critical Care), University of Montreal Hospital, Montréal, QC Canada; 9 Canadian Donation and Transplantation Research Program, Data and Innovation Expert Group, Toronto, ON Canada; 10 Centre hospitalier de l’Université de Montréal Research Center, Université de Montréal, Montréal, QC Canada; 11 Center for Artificial Intelligence in Medicine & Imaging, Stanford University, Stanford, CA USA; 12 Mila, Quebec Artificial Intelligence Institute, Montréal, QC Canada; 13 Canada Excellence Research Chair, Polytechnique Montréal, Montréal, QC Canada; 14 Department Microbiology, Infectiology and Immunology, Faculty of Medicine, Université de Montréal, Montréal, QC Canada; 15 Héma-Québec, Montréal, QC Canada; 16 Division of Gastroenterology and Hepatology, Department of Medicine, University of Toronto, Toronto, ON Canada

**Keywords:** Machine learning, Computational models

## Abstract

Solid-organ transplantation is a life-saving treatment for end-stage organ disease in highly selected patients. Alongside the tremendous progress in the last several decades, new challenges have emerged. The growing disparity between organ demand and supply requires optimal patient/donor selection and matching. Improvements in long-term graft and patient survival require data-driven diagnosis and management of post-transplant complications. The growing abundance of clinical, genetic, radiologic, and metabolic data in transplantation has led to increasing interest in applying machine-learning (ML) tools that can uncover hidden patterns in large datasets. ML algorithms have been applied in predictive modeling of waitlist mortality, donor–recipient matching, survival prediction, post-transplant complications diagnosis, and prediction, aiming to optimize immunosuppression and management. In this review, we provide insight into the various applications of ML in transplant medicine, why these were used to evaluate a specific clinical question, and the potential of ML to transform the care of transplant recipients. 36 articles were selected after a comprehensive search of the following databases: Ovid MEDLINE; Ovid MEDLINE Epub Ahead of Print and In-Process & Other Non-Indexed Citations; Ovid Embase; Cochrane Database of Systematic Reviews (Ovid); and Cochrane Central Register of Controlled Trials (Ovid). In summary, these studies showed that ML techniques hold great potential to improve the outcome of transplant recipients. Future work is required to improve the interpretability of these algorithms, ensure generalizability through larger-scale external validation, and establishment of infrastructure to permit clinical integration.

## Introduction

There has been tremendous progress in the outcomes of solid-organ transplantation in recent decades. Nonetheless, there remain challenges at various levels of the transplant journey. Organ allocation is a major limiting factor as there is constant increased demand while the donor organ supply is limited^1^. In addition, the increasing medical complexity of transplant candidates with their advanced age, metabolic risk factors, and cardiovascular comorbidities is associated with higher risks of morbidity and mortality on the waiting list and after transplant, resulting in higher risk of infections, malignancy, and medication-induced side effects. As a result, allocating organs to appropriate recipients who will benefit the most from transplantation with the lowest possible risk is a significant challenge^2–5^.

The allocation algorithms must consider transplant outcomes that depend on a complex combination of factors including patient demographics, comorbidities, genetics, graft quality, and more. Although short-term outcomes have markedly improved secondary to better surgical techniques, optimization of immunosuppressive therapy and post-operative management, complications due to graft rejection and secondary to long-term use of immunosuppressive medications can result in significant morbidity and mortality^6^. To overcome these issues, protocols for optimizing immunosuppression based on biomarkers are needed, but cannot provide patient-level predictions^7–9^.

Machine learning (ML) is a branch of Artificial Intelligence (AI) in which a computer algorithm learns from examples to generate reproducible predictions and classifications on previously unseen data^10,11^. Machine learning can be (1) supervised, referring to manually mapping an observation’s characteristics to a known outcome; (2) unsupervised, referring to the discovery of innate patterns using unlabeled data; or (3) reinforcement learning, referring to the training of ML models in an interactive environment to make a sequence of decisions by employing trial and error through ongoing feedback^11^. ML can analyze large, complex and heterogeneous datasets, yielding sophisticated outcomes and predictive models. ML techniques have been applied in different fields in medicine where large datasets with complex data points exist, resulting in the generation of important predictive models with the potential to ameliorate clinical practice.

Novel applications of ML techniques in transplant medicine have emerged and are constantly evolving. The prediction of post-transplant outcomes is extremely complex and involves a large amount of clinical, laboratory, genetic, immunologic, and metabolic data. Beyond waitlist prioritization and organ allocation, other areas of application in transplant medicine include a better identification of potential organ donors, prediction of overall survival, short- and long-term complications, and pharmacokinetic analyses^12^.

**Fig. 1:**
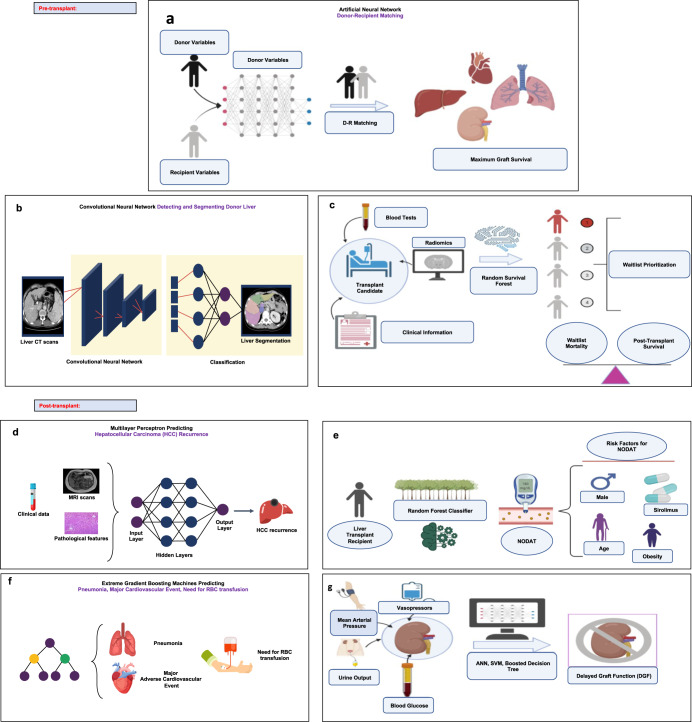
Applications of ML in solid-organ transplantation. **a** Artificial Neural Networks (ANNs) benefit from automatically learning from high-dimensional data and detecting complex nonlinear relationships between input variables and outcome of interest. ANNs report high accuracy in optimal identification of potential organ donors. **b** Convolutional Neural Network (CNNs) are neural network models that are popular for image classification tasks and help in efficient feature extraction through convolution operation and perform efficient segmentation of donor's liver through input data in the form of MRIs. **c** Random Survival Forest (RSF) approach is an Ensemble tree method resulting in better survival prediction and variable selection. Through RSF laboratory and hemodynamic variables affecting waitlist mortality can be identified through interpreting nonlinear relationships between the variables. **d** Multilayer perceptions are neural networks that identify complex nonlinear relationships in the data and can help in handling different data domains such as clinical and image features together to predict Hepatocellular Carcinoma (HCC) recurrence with high accuracy. **e** In Liver transplant recipients, Random Forest (RF) classifier is a tree-based classifier that generalizes classifications using decision trees and can efficiently identify important risk factors relevant to new-onset diabetes after transplantation (NODAT). **f** Gradient boosting machines employ sequential decision trees which reduce the error by training on the error residuals and can classify a subject into a candidate for risk of pneumonia, RBC transfusion etc. so that clinicians can efficiently filter patients requiring immediate support. **g** Important risk factors for Delayed Graft function (DGF) can be provided to ANNS, Support Vector Machine (SVMs) and tree-based models to identify patients at higher risk of DGF. ANNs can be applied on high-dimensional datasets, however, when complexity is low, SVMs and decision trees can provide more interpretable modeling.

In this review, we aim to provide an overview of recent advances, potential power, and limitations of ML applications in transplant medicine. Waitlist prioritization, donor–recipient matching, and post-transplant outcomes are the three main sections of this review. An overview of ML applications in solid-organ transplantation is depicted in Fig. 1.

## Methods

A comprehensive search strategy was initially developed for Medline (Ovid) using a combination of database-specific subject headings and text words for the main concepts of solid-organ transplantation, donation, and machine learning. The search strategy was then customized for each of the other databases. The following databases were searched on September 2020 and limited to years 2015–2020: Ovid MEDLINE; Ovid MEDLINE Epub Ahead of Print and In-Process & Other Non-Indexed Citations; Ovid Embase; Cochrane Database of Systematic Reviews (Ovid); and Cochrane Central Register of Controlled Trials (Ovid).

**Fig. 2:**
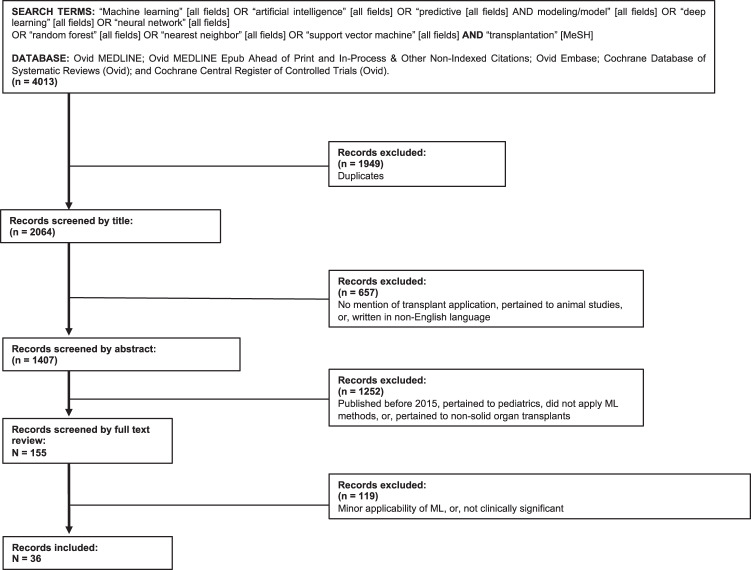
Flowchart of search strategy and selection of studies for inclusion. Database search retrieved 155 papers for initial review. In total, 36 papers were included in the final review according to clinical significance and relevance to machine learning, transplantation, and donation.

The queries retrieved 155 papers for initial review. The citations were reviewed manually by NG, NJS, and AS. In all, 36 papers were included in this review according to clinical significance and relevance to machine learning, transplantation, and donation. The relevant articles which were included in the review are presented in Table 1. The flowchart of this process is illustrated in Fig. 2.

**Table 1:** Machine-learning applications studies in adult solid-organ transplant recipients.

Category	Title	Author(s), year	Patient population	Transplant type	Main outcomes
Organ allocation	Variables of importance in the Scientific Registry of Transplant Recipients database predictive of heart transplant waitlist mortality	Hsich et al.^14^	33,069 waitlist	Heart	An RF model identified predictors of waitlist mortality and was used to produce a risk score. eGFR and serum albumin were identified and not currently considered in the allocation system.
	Simulating the outcome of heart allocation policies using deep neural networks	Medved et al.^15^	30,584 recipients and 18,982 donors	Heart	Organ allocation using Neural network-based algorithm named Lund Deep Learning Transplant Algorithm (LuDeLTA) achieved the predicted post-transplant survival of 4700 days versus 4300 days by allocation using clinical rules
	Detection of potential organ donors; an automatic approach on temporal data	Sauthier et al.^16^	Potential donor pool from ICU	NA	The use of a ANN and a Logistic Regression model trained on 105 distinct laboratory analyses resulted in similar performance with an Area Under the Curve (AUC) of 0.950 (95% Confidence Interval (CI) 0.923–0.974) and 0.947 (95% CI 0.9169–0.9730), respectively
	Ant lion optimization algorithm for kidney exchanges	Hamouda et al.^19^	Simulated dataset	Kidney	An Ant Lion Optimizer-based program achieved comparable kidney exchange results to the deterministic-based approaches like integer programming and outperformed other stochastic-based methods such as Genetic Algorithm in terms of efficiency and the quantity of resulting exchanges.
	Validation of artificial neural networks as a methodology for donor–recipient matching for liver transplantation	Ayllón et al.^21^	822 donor–recipient pairs	Liver	ANN model for D-R matching had an excellent prediction for 3- and 12-month graft survival (AUC = 0.94 and 0.78, respectively), nearly 15% higher than the MELD score.
	Dynamically weighted evolutionary ordinal neural network for solving an imbalanced liver transplantation problem	Dorado-Moreno et al.^22^	634 donor–recipient pairs	Liver	A decision-support tool was developed based on ANNs to inform organ allocation. The best model yielded an accuracy of 73%, with the geometric mean of the sensitivities of 31.46%, outperforming current state-of-the-art models.
	Can donor narratives yield insights? A natural language processing proof-of- concept to facilitate kidney allocation	Placona et al.^23^	74,041 donors	Kidney	Natural Language Processing model was used to predict the delay or discard of adult deceased donors based on donor-free-text data (C-statistic = 0.75). Performed on par with traditional methods. New clinical and social variables were identified to affect kidney utilization.
	Development of a Predictive Model for Deceased Donor Organ Yield	Marrero et al.^24^	75,350 recipients	Multi	Various ML and traditional models were applied to predict donor organ yield. BART performed with the lowest in error (*P* < 0.001, MAE = 0.856).
Survival	Improving prediction of heart transplantation outcome using deep learning techniques	Medved et al.^25^	27,705 recipients	Heart	2 models predicting 1-year post-transplant mortality were compared. International Heart Transplantation Survival Algorithm (IHTSA), developed using deep learning technique, yielded an ROC = 0.654 (95% CI: 0.629–0.679) and Index for Mortality Prediction After Cardiac Transplantation (IMPACT) yielded an ROC = 0.608 (95% CI: 0.583–0.634).
	Personalized survival predictions via Trees of Predictors: An application to cardiac transplantation.	Yoon et al.^26^	51,971 recipients, 30,911 waitlist	Heart	Developed Trees of Predictors (ToPs) method; improved accurate prediction of survival in heart transplants across all assessed time intervals of post-transplant survival evaluation (AUC = 0.660) compared to existing clinical risk scores (AUC = 0.587).
	Predictive Abilities of Machine-Learning Techniques May Be Limited by Dataset Characteristics: Insights From the UNOS Database.	Miller et al.^27^	56,477 recipients	Heart	Compared ML and traditional statistical techniques predicting 1-year survival post heart transplant. ML models (neural networks, naive Bayes, tree-augmented naive Bayes, support vector machines, random forest, and stochastic gradient boosting) showed similar discrimination capabilities with traditional models (logistic and ridge regression) (C-statistic ≤0.66, all). ML models can be limited by dataset quality.
	Using machine learning and an ensemble of methods to predict kidney transplant survival	Mark et al.^28^	163,199 observations	Kidney	An ensemble of random survival forests and Cox proportional hazards model had a 5-year concordance index of 0.724 vs 0.697 obtained by Estimated Post-Transplant Survival (EPTS) model
	A Machine-Learning Approach Using Survival Statistics to Predict Graft Survival in Kidney Transplant Recipients: A Multicenter Cohort Study	Yoo et al.^31^	3117 recipients	Kidney	Compared conventional and ML methods (decision tree/cox hazard vs survival decision tree, ridge, LASSO, bagging, random forest) to predict graft survival; a survival decision tree yielded the highest C-index of 0.80. Acute rejection within the 1st year is associated with a 4.27-fold increase in the risk of graft failure in the future.
	Prediction of Perioperative Mortality of Cadaveric Liver Transplant Recipients During Their Evaluations	Molinari et al.^32^	30,458 recipients	Liver	ML methods to identify predictors of mortality 90 days post transplant. A scoring system was created based on these predictors (MELD, BMI, age, diabetes, pre-transplant dialysis) resulting in a model with AUC = 0.952 for the discrimination of patients with 90-day mortality risk at ≥10%.
	Training and Validation of Deep Neural Networks for the Prediction of 90-Day Post-Liver Transplant Mortality Using UNOS Registry Data	Ershoff et al.^33^	57544 recipients	Liver	Comparing models predicting mortality 90-day post LT, a DNN model (AUC = 0.703, 95% CI: 0.682–0.726) did not result in higher discriminative performance compared to the SOFT score (AUC = 0.688, 95% CI: 0.667–0.711).
	Five Years Survival of Patients After Liver Transplantation and Its Effective Factors by Neural Network and Cox Proportional Hazard Regression Models	Khosravi et al.^34^	1168 recipients	Liver	ANN model outperformed Cox PH model predicting 5-year survival post-transplant (AUROC = 86.4% and 80.7%, respectively).
	Identifying the Prognosis Factors in Death after Liver Transplantation via Adaptive LASSO in Iran	Raeisi Shahraki et al.^35^	680 recipients	Liver	LASSO was compared with ridge regression methods in identifying predictors of mortality. LASSO resulted in AUC = 89% (95% CI: 86–91%) and significantly outperformed traditional methods (*P* < 0.001).
	Identifying Factors That Affect Patient Survival After Orthotopic Liver Transplant Using Machine-Learning Techniques	Kazemi et al.^36^	902 recipients	Liver	3 step feature selection method predicting LT survival using an SVM classifier resulted in AUC = 0.90, sensitivity = 0.81.
Rejection/graft failure	Machine-Learning Algorithms Predict Graft Failure After Liver Transplantation	Lau et al.^37^	180 recipients and donors	Liver	To better predict graft failure, ML methods were used to identify the top 15 donor and recipient characteristics. Random forests utilizing these 15 factors resulted in AUROC = 0.818 (95% CI: 0.812–0.824) serving as a good proof-of-concept. This was better than that of the Donor Risk Index and Donor Risk Index combined with MELD scores.
	A neural network approach to predict acute allograft rejection in liver transplant recipients using routine laboratory data	Zare et al.^38^	148 recipients	Liver	Used feed-forward, back propagation neural network-based model to predict acute liver rejection. AST and ALT were found to be the most important predictors. The model’s accuracy was 90%, sensitivity was 87%, and specificity was 90% in the testing set, outperforming LR.
	Prediction of Kidney Graft Rejection Using Artificial Neural Network	Tapak et al.^39^	378 recipients	Kidney	Compared ANN to LR in identifying risk factors for chronic non-reversible rejection. ANN outperformed LR (AUC = 0.88 and AUC = 0.75, respectively). Predictive variables included recipients’ age, creatinine level, cold ischemic time, and hemoglobin level at discharge.
	Immune Profiles to Predict Response to Desensitization Therapy in Highly HLA-Sensitized Kidney Transplant Candidates	Yabu et al.^40^	20 recipients	Kidney	Used decision trees and SVM to analyze baseline/longitudinal immune profiles; combining in a multivariate analysis produced seven variables of importance in predicting response to desensitization therapy.
	A Novel CNN-Based CAD System for Early Assessment of Transplanted Kidney Dysfunction	Abdeltawab et al.^42^	56 recipients	Kidney	Computer-aided diagnostic system incorporating CNN for early detection of acute renal transplant rejection, using both clinical and imaging biomarkers. Accuracy of the proposed system = 92.9%, sensitivity = 93.3%, specificity = 92.3% specificity.
	An integrated molecular diagnostic report for heart transplant biopsies using an ensemble of diagnostic algorithms	Parkes et al.^43^	454 recipients	Heart	Microarray data from endomyocardial biopsies were analyzed using supervised binary classifiers to identify molecular rejection (AUC > 0.87) and outperformed histologic rejection (AUC < 0.78), even when trained on a histologic diagnosis.
	Molecular assessment of rejection and injury in lung transplant biopsies	Halloran et al.^44^	209 recipients	Lung	Microarray assessment using single-piece transbronchial biopsies (TBBs) identified four phenotypes: normal, T-cell mediated rejection, antibody-mediated rejection, and injury.
	Molecular phenotyping of rejection-related changes in mucosal biopsies from lung transplants	Halloran et al.^45^	214 recipients	Lung	Mucosal biopsies were used for microarray and expression analysis using unsupervised ML methods. Rejection was associated with IFNG-inducible transcripts
	Use of a Targeted Urine Proteome Assay (TUPA) to identify protein biomarkers of delayed recovery after kidney transplant	Williams et al.^46^	52 recipients	Kidney	A Targeted Urine Proteome Assay used to collect biomarkers of delayed graft function in for early identification, which were analyzed by an iterative Random Forest analysis. Four proteins were highlighted and resulted in a sensitivity of 77.4% and specificity of 82.6% (AUC = 0.891).
	The impact of deceased donor maintenance on delayed kidney allograft function: a machine-learning analysis	Costa et al.^47^	443 recipients	Kidney	Using ML methods, mean arterial pressure, >1 or high dose vasopressors and blood glucose were identified as risk factors of delayed graft function, which were not detected using multivariate logistic regression. Top performing models were boosted DT using C5.0 algorithm (AUC = 0.791), boosting NN (AUC = 0.886), and SVM with polynomial kernel (AUC = 0.784).
Post-transplant complications	Evolution and Determinants of Health-Related Quality-of-Life in Kidney Transplant Patients Over the First 3 Years After Transplantation	Villeneuve et al.^48^	337 recipients	Kidney	A K-means method was used to identify 2 clusters in longitudinal quality-of-life data. Covariates from this were analyzed with random forest methods. Muscular weakness and anxiety 1-year post transplant attributed to 19 and 24% respectively, to variability in quality- of-life scores among patients at 3 months post transplant
	Archetype Analysis Identifies Distinct Profiles in Renal Transplant Recipients with Transplant Glomerulopathy Associated with Allograft Survival	Aubert et al.^49^	385 recipients	Kidney	An unsupervised learning method integrating clinical, immune, and outcome variables revealed five transplant glomerulopathy archetypes characterized by distinct functional, immunologic, and histologic features. These archetypes were associated with distinct causes and allograft survival profiles.
	Prediction of Acute Kidney Injury after Liver Transplantation: Machine-Learning Approaches vs. Logistic Regression Model	Lee et al.^54^	1211 recipients	Liver	ML methods (decision tree, RF, gradient boosting machine, SVM, naive Bayes, multilayer perceptron, and deep belief networks) were compared with logistic regression to predict AKI post-transplant. Gradient boosting machine yielded the best performance in AUROC to predict AKI of all stages (0.90, 95% CI: 0.86–0.93). Logistic regression produced 0.61 (95% CI: 0.56–0.66).
	Computer-assisted liver graft steatosis assessment via learning-based texture analysis	Moccia et al.^50^	40 donors	Liver	RBG photos of grafts were analyzed along with blood sample parameters using supervised & unsupervised learning for feature classification. The best model yielded classification sensitivity = 0.95, specificity = 0.81, and accuracy = 0.88.
	New-Onset Diabetes and Pre-existing Diabetes Are Associated With Comparable Reduction in Long-Term Survival After Liver Transplant: A Machine-Learning Approach	Bhat et al.^51^	61,677 recipients	Liver	Random forest classifier identified male sex, obesity, and older age are risk factors of NODAT. 10-year long-term survival was similar between those with pre-existing diabetes vs. NODAT.
	Decision tree analysis to stratify risk of de novo non-melanoma skin cancer following liver transplantation	Tanaka et al.^52^	105,984 recipients	Liver	A decision tree analysis was used to stratify risk factors of developing de novo non-melanoma skin cancer. Caucasian males >47 years, with BMI <40, and who did not receive sirolimus, were identified as high risk (7.3% cumulative incidence of NMSC).
	Predicting Low Risk for Sustained Alcohol Use After Early Liver Transplant for Acute Alcoholic Hepatitis: The Sustained Alcohol Use Post-Liver Transplant Score	Lee et al.^53^	134 recipients	Liver	Logistic and Cox regression, CARTs, and LASSO regression were used to identify predictors of sustained alcohol use post transplant, which was then the basis of a new scoring system (SALT) (C-statistic = 0.74 in internal cross-validation dataset).
	Machine-Learning Algorithms Utilizing Quantitative CT Features May Predict Eventual Onset of Bronchiolitis Obliterans Syndrome After Lung Transplantation	Barbosa et al.^56^	71 recipients	Lung	Applied SVM on qCT data to identify profiles of Bronchiolitis Obliterans Syndrome post transplant. An accuracy of 0.85 using three qCT parameters.

### Organ allocation and predictive modeling for waitlist mortality

Many existing organ allocation policies hinge on a few criteria based on recipient need for transplant and donor–recipient matching. An optimal allocation system should incorporate important factors affecting waitlist mortality by expansion of the data types considered. ML may be a suitable tool for this task by identifying variables of importance from both recipients and donors and be utilized for the assessment of complex nonlinear interactions. Random Survival Forest (RSF) approach is an Ensemble tree method for analysis of right-censored survival data resulting in better survival prediction and variable selection through bagging of classification trees^13^. For example, the RSF approach was applied on a dataset of 33,069 patients waiting for heart transplant, and nine laboratory and hemodynamic variables affecting waitlist mortality were identified^14^. Two of these variables (eGFR and serum albumin) are not currently considered in the United Network for Organ Sharing (UNOS) allocation system (i.e., 6-tiered heart allocation system), and RSF further identified nonlinear relationships between these variables. Furthermore, RSF showed that the importance of each variable correlated with their effect on other variables. For example, in patients with eGFR >40 mL/min/1.73 m^2^, RSF found sex differences as a predictor of waitlist mortality. The advantage of RSF is the identification of predictive variables for waitlist mortality without prior knowledge of parametric relationships. It can manage various interactions and significant missingness unlike conventional statistical methods such as Cox proportional hazards models. However, the model was trained on the Scientific Registry of Transplant Recipients (SRTR) database which only collects certain variables at specific timepoints and has a certain degree of missingness.

Other ML studies have merged both donor and recipient characteristics to optimize post-transplant outcomes rather than waitlist mortality. Artificial Neural Network (ANN) models are used for nonlinear modeling of the input features through a collection of neurons that take input and, in conjunction with information from other nodes, develop output without programmed rules. An ANN-based model was developed to predict waitlist mortality, post-transplant survival, and to simulate the heart allocation process. The model was trained on both donor and recipient data and was able to predict waitlist and post-transplant mortality with significant accuracy (AUROC = 89% and AUROC = 66%, respectively)^15^. This study showed that transplant allocation using ANNs was able to utilize 124 more available hearts compared to the cox regression model. Accordingly, ANNs have been shown to be efficient in organ allocation where prognosis depends on the complex interaction between multiple variables pertaining to both donor and recipient.

In summary, ANNs and RSF models provide better accuracy and deal well with nonlinear interactions in the data while predicting waitlist mortality as compared to other conventional approaches.

### Optimization of donor–recipient matching

#### Donor identification and matching

Potential organ donor identification relies entirely on timely identification of these patients and their referral to Organ Procurement Organizations (OPOs). More optimal identification of potential organ donors may be feasible using ML. A recent study demonstrated that both ANN and logistic regression models trained on 105 distinct laboratory test variables from 19,717 ICU patients resulted in similar performance with an area under the curve (AUC) of 0.950 (95% CI 0.923–0.974) and 0.947 (95% CI 0.9169–0.9730), respectively^16^. However, in this study, the ANNs accuracy was more consistent across different subgroups compared to the logistic regression model which obtained lower AUC for non-referred potential organ donors (AUC for ANN: 0.95 versus LR: 0.78). ANNs benefit from automatically learning from high-dimensional data and detecting complex nonlinear relationships between input variables and ultimate outcome of interest^17^. However, ANNs require large data to be trained on in order to estimate their predictive parameters accurately. On the other hand, logistic regression method benefits from a small number of hyperparameters in its modeling but is not suitable to capture nonlinear relationships in its conventional form, wherein it assumes linearity between the dependent variable and the independent variables^18^.

Donor selection is a challenging and multifactorial decision influenced by both donor and recipient factors as well as match considerations. Risk models have been used in the heart, kidney, and liver transplantation, to better assess the interactions between donor and recipient factors and their overall impact on post-transplant outcomes. Accordingly, ML and discrete optimization have been used in the context of paired kidney exchange^19^. The aim of the kidney exchange program is to obtain the maximum number of donor–recipient matches in a pool of incompatible pairs^19^. For this purpose, a stochastic-based ML algorithm (an algorithm that can make use of randomness during learning) called Ant Lion Optimization (ALO) using the bio-inspired technique was proposed, having the advantage of requiring relatively small computing power in consideration for the typical resources accessible by hospitals and was successful in generating possible pair matches. Its performance was on par with deterministic-based models such as integer programming and outperformed other stochastic-based models including Genetic algorithm^19^. This algorithm was also able to give higher weight to patients who had lower chances of getting matched by conventional model^19^.

ML tools have been used in the integration of specific donor characteristics with recipient ones to produce matches with maximal post-transplant survival. In liver transplantation, a study of 822 donor–recipient pairs from King’s College Hospital (London, UK) successfully validated an ANN model that originally had been developed using multicentric Spanish cohorts^20^. This ANN model was trained to help clinicians with donor–recipient matching to achieve maximum graft survival at 3 and 12 months after liver transplant^21^. The ultimate outcomes of interest were graft survival and non-survival at three (AUC 0.94 for ANN) and twelve months (AUC 0.78 for ANN). Finally, ANN achieved 20% higher AUC compared to Model For End-Stage Liver Disease (MELD) score in predicting 3-month graft survival^21^. The number of days on waitlist, underlying liver disease, and MELD score were among most predictive variables from recipient and cause of death, cold ischemic time, hypertension and AST were among top-ranked features from donor side for predicting 3-month graft survival. Although the ANN model performed even better using data from the English center compared to the original Spanish cohorts, it is still necessary to optimize and fine-tune the model when new data are available from other transplant units. Also, using the same ML model in countries with different health care systems may not achieve the same significant performance due to the heterogeneity and different variables collected in various programs^21^. In another study, an ANN-based algorithm trained on data from seven Spanish hospitals and one hospital from the UK correctly predicted liver transplant outcomes for 73% of the population. The ANN algorithm was trained to predict the risk of graft failure within 15 days, between 15 and 90 days, and over 90 days after transplant based on the features from both the donors and the recipients. Compared to ANN (with 73% correct prediction), logistic regression, and SVM models showed correct prediction only for 50 and 66% of the population, respectively^22^. However, this ordinal classification of post-transplant outcome resulted in imbalanced subgroups with more than 85% of recipients surviving more than 90 days after LT. The authors solved this imbalance challenge by giving dynamic weights to the minority classes by using a cost-sensitive evolutionary ordinal artificial neural network and an ordinal oversampling technique. This ANN algorithm can serve as a decision-support system beside MELD score and other clinical risk scores for transplant hepatologists to make more informed decisions based on both donor and recipient characteristics.

#### Decision-support tools for organ donation

While there is a high demand versus supply for donor organs, some organs are still discarded in the process. Marginal grafts have unpredictable acceptance rates which vary between centers but are offered to all centers based on need and regardless of their capabilities to perform transplant for hard-to-place organs. Decision-support tools that identify deceased donor kidneys which may experience placement difficulties and streamline this process may increase donor organ utilization. To better assist in accept/decline decisions for patients needing adult kidney donors, Natural Language Processing (NLP) methods that tap into free-text data beside structured data from donor information were studied^23^. Using this method, both known and new key clinical terms holding significant predictive value were discovered, producing a model with a C-statistic of 0.75 for accept or decline decisions which is comparable with the performance of traditional indices including Reduced Probability of Delay or Discard (r-PODD, C-statistics = 0.80) and Kidney Donor Profile Index (KDPI, C-statistics = 0.77). Intravenous drug use as well as some other keywords pertaining to cardiovascular disease such as “stent”, “CHF”, and “cholesterol” in the free text were found among predictive words for discarding donor organs using the NLP method. However, these variables are not documented in structured data from OPTN. Accordingly, a combination of structured (from OPTN database) and unstructured data (from free text) can improve the performance of ML models for this purpose. Another study of 75,350 deceased donors from OPTN database comparing linear statistical models with ML algorithms in prediction of organ yield from a deceased donor. Organ yield was defined as the number of organs transplanted per donor. This study showed that Bayesian Additive Regression Trees (BART) had the highest yield with the lowest Mean Absolute Error (*P* < 0.001) and highest resolution out of 13 models in predicting deceased donor organ yield^24^. BART is a nonparametric Bayesian regression approach that creates a binary tree by recursively splitting the data on the predictor values using a statistical model consisting of a prior and a likelihood. Advantages for BART include easier pre-processing and visualization of data and capturing nonlinear relationships during prediction whereas, disadvantages include high model complexity and instability in the tree in case of small changes in data. Accordingly, these studies suggest how ML can facilitate the identification/availability of potential donors.

#### Strengths and weaknesses of machine-learning algorithms in pre-transplant settings

ML models hold great potential in helping clinicians with the prediction of waitlist mortality, organ allocation, donor–recipient matching, and donor organ assessment. The main strength of these models is their capacity to work efficiently with large datasets, and to find complex hidden relationships between donor and recipient characteristics, leading to better performance compared to conventional statistical algorithms such as logistic regression. However, the main limitation of ML models is their dependence on the quality of input data especially in large data registries which are susceptible to human error in data documentation. Moreover, heterogeneity and variations in collected data between different transplant centers require clinicians to fine-tune ML models using the variables from their local data registry. Moreover, since these algorithms work better with large datasets, they also require systems with high computing power for data analysis which may not be accessible in every clinical setting.

### Post-transplant outcomes

#### Prediction of post-transplant survival

Optimal decision-making and management rely on prediction of patient survival on the waiting list and after transplant, aiming to increase the number of successful transplants and improve overall outcome. Several survival models before and after transplant have been built using deep learning techniques.

The International Heart Transplantation Survival Algorithm (IHTSA) model was an ANN-based model derived and tested from a pool of 27,705 adult patients from the UNOS registry, utilizing both recipient and donor variables. The model outperformed a conventional logistic regression-based model (i.e., Index for Mortality Prediction After Cardiac Transplantation (IMPACT)) in accurate prediction of 1-year mortality (AUC 0.654 vs. 0.608, *P* = 0.004) and long-term survival (C-index 0.627 vs 0.584)^25^. The added capabilities of deep learning in capturing nonlinear and hidden patterns resulted in error reductions by 12% in prediction of short-term mortality and by 10% in long-term mortality when compared with the traditional models. This survival model consists of a flexible nonlinear generalization of the standard Cox proportional hazard model, integrating ensembles of ANNs with a prediction capability of more than 1 year. Although the IHTSA model includes both donor and recipient variables compared to the IMPACT model which only includes recipient variables, IHTSA still showed significantly better performance even after training using similar variables with the IMPACT model. This also confirms that ANN-based models benefit from identifying new patterns among the same input variables compared to conventional models^25^.

Similarly, a decision tree ML algorithm including 53 donor, recipient, and donor–recipient compatibility features for heart transplant candidates resulted in improved prediction of survival across all assessed time intervals of post-transplant survival evaluation compared to risk-stratification score (RSS)^26^. The decision tree algorithm predicted 3-year survival correctly for 14% more patients compared to RSS after holding specificity at 80%. This ML model overcame the challenge of heterogeneity in this patient population by identifying clusters with similar features among the whole patient population and finding the specific predictive variables for survival for each cluster^26^. This suggested that ML-derived methods may be better able to adapt and outperform conventional models despite changes in clinical practice over time. However, a separate study on survival prediction using pre-transplant variables did not appreciate any improvement in 1-year post-transplant survival prediction when comparing standard statistical methods (C-statistic = 0.65) to a myriad of ML methodologies (C-statistic = 0.66 for ANN)^27^, despite adjusting for policy changes in allocation over time. The handling of missing data within the UNOS database differed between the two above studies, suggesting that data quality and management could potentially play a significant role in the resultant predictability of ML models.

In kidney transplantation, the use of a combination of Random Forest (RF) classification and Cox regression with least absolute shrinkage and selection operator (LASSO) for variable selection on 73 donor and recipient characteristics resulted in a model that outperformed the standard Estimated Post-Transplant Survival (EPTS) model for 5-year survival prediction (concordance index 0.724 vs. 0.697)^28^. They trained two separate models for recipients below and above 50 years old and finally obtained two different sets of predictive variables for these two cohorts^28^. Accordingly, this study showed that developing separate ML models for different cohorts of patients may improve their accuracy even further. LASSO regression technique uses regularization to avoid overfitting but leads to arbitrary dropping of the predictors when those are highly correlated^29^. RF technique on the other hand, combines decisions from multiple decision trees, making it highly flexible and accurate while estimating nonlinear relationships in the data^30^. These survival decision trees have also been used to predict kidney graft survival, outperforming conventional statistical methods (such as cox regression model and conventional decision tree model), as seen in a study of over 3000 renal transplant patients^31^. These models obtained a 3-month serum creatinine level as one of the most important features for predicting graft failure. The survival decision tree model was able to take advantage of censored patient data and to add interpretable clinical information using survival statistics^31^.

ML methodologies are perhaps the most well studied in liver transplant recipients. Two studies utilizing preoperative UNOS data from adult transplant recipients for the assessment of 90-day mortality separately highlight the importance of feature selection^32,33^. Pre-selected variables of recipient age, BMI, MELD score, history of preoperative renal replacement therapy and diabetes were used to develop an ML-based scoring system with an AUC of 0.952 in identifying high-risk patients, defined as those with predicted probability of death greater than 10%^32^. Similar studies using ANNs and LASSO regression have been performed on smaller patient populations to effectively predict post-transplant survival^34–36^. RF predicted post-operative graft failure in the immediate post-operative period up to 30 days with greater accuracy than Logistic Regression and scoring indices such as the Donor Risk Index (DRI) and the survival outcomes after liver transplantation (SOFT) score, with AUROC = 0.818 (95% CI: 0.812–0.824)^37^. Furthermore, ANN algorithm trained on the top 15 important pre-transplant features outperformed RF (AUC: 0.835 vs 0.818), showing the importance of feature selection to improve the model performance^37^.

#### Prediction of rejection

Several studies aimed to predict rejection and identify those at greatest risk. These studies however have been limited in their ability to accurately ascertain risk. Given the often-large number of contributory factors to rejection, ML may play a role in managing many data points, considering not only readily available clinical data variables but also genetic, metabolomic, and pathology-based variables that may not have been previously utilized for model development to provide better prediction capability. ANNs have consistently been proven to be highly accurate predictive tools for graft rejection in both renal and liver transplantation compared to standard modeling techniques^37–39^.

In liver transplantation, ANNs outperformed logistic regression models in predicting the risk of acute rejection in 148 recipients using clinical and laboratory test data at 7 days after transplant, with 90% accuracy, 87% sensitivity, and 90% specificity^38^.

In renal transplantation, sensitization defined as the formation of antibodies against human leukocyte antigens (HLA) makes successful transplant for highly sensitized candidates difficult. Many desensitization processes exist; however, patients often fail to respond to therapy, and it can be difficult to determine which factors make one patient an appropriate candidate for desensitization therapy. Through evaluation of various assays of immune and gene expression profiles of highly sensitized patients, a Support Vector Machine (SVM) model was able to identify a distinguishing pattern for those likely to respond to desensitization therapies^40^. SVM classification is based on finding a hyperplane in a high-dimensional space representing the largest margin that distinctly classifies the data points of the two outcomes^41^. The dimension of the hyperplane depends upon the number of features, if two the hyperplane is just a line and if there are three features, then the hyperplane becomes a 2-D plane and so on. SVMs are robust to overfitting, however, do not scale well to large datasets. Utilization of ML processes was able to highlight variables of importance for classification and also allowed for the identification of important patterns that would have otherwise been difficult to ascertain with conventional methodology.

ML methodology can also be advantageous in allowing for boosted diagnostic performance through simultaneous assessment of varied data types. In a study combining data obtained from diffusion-weighted MRI images and clinical biomarkers such as creatinine clearance and serum plasma creatinine, a Convolutional Neural Network (CNN) was able to accurately identify 92.9% of rejected kidney grafts regardless of scanner type and differences in image collection protocols between patients^42^. CNNs are neural network models that are popular for image classification tasks and help in efficient feature extraction through convolution operation when studies consist of clinical imaging and radiomics features such as MRI data as input^17^. Through this approach, deep learning tools combining medical image analyses with clinical data highlight the opportunities for early, noninvasive detection of rejection in solid-organ transplantation as an alternative for graft biopsy.

The diagnosis of graft rejection is historically made by tissue histopathology assessment. However, graft biopsy assessment is usually limited by low reproducibility and inter-observer variability. In attempts to mitigate the variability in interpretation of biopsy results, ML has been utilized to provide more definitive, standardizable methods. Supervised learning methods have been used with endomyocardial biopsies of heart transplant recipients to predict rejection using microarray analysis^43^. Accordingly, molecular classifiers enabled better molecular rejection prediction than histologic rejection (AUCs >0.87 compared to AUCs <0.78, respectively). The authors also utilized an automated RF model which was highly predictive of corresponding expert diagnoses based on the molecular reports suggesting that these algorithms could be utilized in lieu of pathologist assessment so as to increase efficiency in diagnosis^43^. Similar microarray analyses have been utilized in lung transplant recipients for molecular phenotyping of rejection from both transbronchial and mucosal biopsies, for which unsupervised learning methods were able to identify molecular signatures correlating with patterns of rejection from samples that would have been previously deemed unusable^44,45^.

### Post-transplant complications

#### ML and kidney transplant complications

In the context of kidney transplantation, delayed graft function (DGF) is defined as the need for dialysis within the first week after transplant, is associated with a higher risk of graft loss in the long term, prolonged hospital stays, and thus costs. ML algorithms can uncover potentially useful prognostic indicators as well as targets for future diagnostic and therapeutic studies.

The RF approach was used to identify urine proteins with predictive value for DGF using targeted urine proteome assay^46^. Data from 52 patients with intermediate, slow, and delayed graft function and urine samples were collected within 12–18 h post-surgery. Four key urine proteins were found to be changed in recipients with DGF, with a sensitivity of 77.4% and a specificity of 82.6% (AUC 0.89). Alternative ML strategies were used to investigate how pre-transplant donor maintenance practices contribute to the onset of DGF. This multicenter study used data from 443 deceased donors. An initial multivariate logistic regression model was not able to identify significant variables^47^. In a post hoc analysis, predictive models that use ML tools, including boosted Decision Tree (DT) using the C5.0 algorithm (AUC 0.79), boosting ANN (AUC 0.88), and SVM with polynomial kernel (AUC 0.78), were able to identify key donor maintenance variables for DGF. The models were also able to recognize nonlinear relationships between variables. This study showed the importance of donor maintenance variables such as urine output and mean arterial pressure which were not included in other regression-based risk scores for predicting post-transplant DGF. However, with its retrospective nature, the data collected were not time-sensitive and may not represent the full picture in terms of donor kidney maintenance. The RF approach was also used to evaluate the health-related quality of life (HRQOL) and its determinants within the first 3 years in 337 kidney transplant recipients. This approach found a significant association between HRQOL at 1 month after transplantation and HRQOL between 3 and 36 months after transplantation^48^. Also, in contrast to conventional models, the ensemble of ML algorithms allowed analyses of both quantitative and qualitative variables together without limitation of the covariates tested^48^.

Another multicenter study applied probabilistic unsupervised classification techniques called archetype analysis to risk-stratify five distinct groups of patients (archetypes) with transplant glomerulopathy although all of them had similar histological morphology (double contour of the glomerular basement membrane)^49^. The study consisted of kidney biopsies from 385 recipients with confirmed glomerulopathy. These archetypes were classified based on post-transplant clinical, functional, immunologic, and histologic data, and were shown to have significantly distinct graft outcomes. These were used to produce an online application which can potentially be used in clinical contexts to flag those kidney transplant recipients with glomerulopathy who are at higher risk of graft failure. The study was successful in using an unbiased approach to classify heterogeneous data, and further studies should elucidate the driving mechanisms of the underlying pathology to understand the produced archetypes.

#### ML and liver transplant complications

Before a liver transplantation, one of the key features that can help surgeons with predicting post-transplant graft function is the amount of graft steatosis. Since biopsy and histological methods of steatosis assessment are expensive and inefficient, physicians are left to use donor clinical characteristics and visual analyses of the graft. The subjectivity and variability of this process remains a challenge, to which researchers employed ML tools on photographic data to detect hepatic steatosis. Using a semi-supervised classification approach on 40 liver images obtained by smartphone camera in the operating room translating to 600 liver patches as well as clinical variables and blood sample tests, an SVM model was able to qualitatively assess grafts steatosis before transplantation with an accuracy of 0.88, sensitivity of 0.95 and specificity of 0.81 considering liver biopsy as the reference method^50^. However, due to the small sample size they used leave-one-patient-out cross-validation for the evaluation of their algorithms which may lead to overestimation of the performance.

Metabolic side effects of immunosuppressive medications including diabetes mellitus compromise the long-term survival of solid-organ transplant recipients. Accordingly, in a study for the prediction of new-onset diabetes after transplantation (NODAT) within the first year after transplant, a RF classifier was able to robustly identify the most important risk factors for NODAT using the SRTR database^51^. This study identified sirolimus-based immunosuppression as a risk factor for NODAT. However, since the SRTR database does not include data about immunosuppressive medication serum levels and fasting glucose, it was not possible to accurately evaluate the association of sirolimus with hyperglycemia. Another common side effect of long-term immunosuppression is de novo non-melanoma skin cancers (NMSC) including basal cell carcinoma (BCC) and squamous cell carcinoma (SCC). This elevated risk begs early detection and prediction methods to allow for timelier adjustments in care. A study conducted by Tanaka & Voigt adopted a decision tree approach to stratify patients based on the risk of developing NMSCs^52^. First, using a cox regression analysis, main independent risk factors including BMI, not receiving sirolimus, and recipient age was identified and fed into the decision tree analysis. Then, these risk factors were ranked based on their importance using decision tree analysis, and various value ranges were defined to stratify patients from low to high risk (validation set: *R*^2 ^= 0.971, *P* < 0.0001). This study showed that recipients with BMI < 40 kg/m^2^ and over 47 years were at higher risk of NMSC and may benefit from more frequent cancer screening compared to other recipients who undergo annual screening for skin cancers. As this study showed, compared to other ML methods, decision trees are more interpretable; this model is able to provide clinicians with guiding algorithms for their clinical management based on the identified risk factors and their cut-offs for different post-transplant outcomes.

Another study developed a novel scoring system using the LASSO regression method to predict the risk of sustained alcohol consumption post-transplant for patients with alcohol hepatitis using to prioritize those candidates with lower risk of relapse for early transplantation. This study used data from 134 liver transplant recipients and the resulting model had a C-statistic of 0.76, after internal cross-validation^53^. Four objective pre-transplant variables including greater than ten drinks per day at initial hospitalization, history of illicit substance abuse, history of any alcohol-related legal issues, and history of multiple rehabilitation attempts were identified as the main predictive risk factors for sustained alcohol consumption after transplant. Accordingly, the LASSO regression method was shown to work well when the number of events is low. More importantly, this model can be used for building risk scores based on the provided coefficients, leading to easier and more explainable implementation of this algorithm in clinical practice. This model is simple to implement and has the potential to open up organ access for those traditionally restricted, however larger and prospective studies would be needed for validation.

Some studies have also directly compared how traditional statistics perform against ML methods. In the context of predicting acute kidney injury (AKI) after liver transplantation, one study compared Logistic Regression to several ML and neural networks models^54^. Models were developed using pre- and perioperative factors from a single-center dataset of 1211 cases with both living and deceased donors. Gradient Boosting yielded the best AUC value (0.90, 95% CI 0.86–0.93) in predicting stages of AKI and performed better than logistic regression methods (AUC 0.61, 95% CI 0.56–0.66). Gradient Boosting builds a sequential series of decision trees, where each tree corrects the residuals in the predictions made by the previous trees. This ML technique is robust when the data contains correlated features, however, this methodology requires tuning of many hyperparameters therefore, rendering model development slower^55^.

#### ML and lung transplant complications

Among lung transplant recipients, chronic lung allograft dysfunction (CLAD) affects more than 50% in the first 5 years post-transplant, and negatively impacts long-term survival. Bronchiolitis obliterans syndrome (BOS) is an obstructive form of CLAD due to chronic immune-mediated rejection. BOS can lead to decreased airflow represented by reduced forced expiratory volume in the first second (FEV1). Early diagnoses can pave the way for future research in interventions. To this end, an SVM to analyze quantitative CT imaging data from 71 patients was investigated as a diagnostic tool for early CLAD detection^56^. Using quantitative CT scan at first post-transplant visit, SVM algorithm was able to identify those recipients who are at higher risk of developing BOS in near future while pulmonary function tests did not show any significant changes in early stages. The model resulted in an 85% accuracy rate using three features from patient images. SVM model identified smaller lobar and airways volumes, smaller airways surfaces, and higher airways resistance as predictive factors for BOS at early quantitative CT scan. Earlier identification of these patients can guide clinicians with appropriate management to avoid further progression of this pathology.

#### Strengths and weaknesses of machine-learning algorithms in post-transplant settings

For post-transplant complications, ML techniques such as SVMs, Random Forests and ANNs have the ability to model evolving and heterogeneous patient-level data over time, identify predictors of poor outcomes, and inform care. Uses of ML in predicting post-transplant complications have numerous applications including decision-support systems and even the development of data-driven assessments. CART and LASSO regression models can guide post-transplant patient management by providing clinicians with transparent algorithms that can be easily understood and explained from a clinical perspective. Moreover, recent research also strives to make ML models more interpretable in terms of identifying features playing a key role during the prediction tasks. Some recent methods including SHapley Additive exPlanations (SHAP) as used for evaluating feature importance and explain the predictions made by ML algorithms toward post-LT AKI^57^, Local Interpretable Model-Agnostic Explanations (LIME) used to assess relative impact of key predictors in post-transplant patient survival^58^, and integrated gradients used to identify important predictors in diagnosing allograft rejection^59^, make ML models more explainable. However, further validation of their results using multicenter prospectively collected data is important before wider application of these algorithms in daily clinical practice.

## Limitations of ML in transplant medicine

While studies show promise for ML; data quality, small sample size, inconsistency regarding the number of cases adequately powering these models and inter-center variability may affect model generalization. Inconsistent data collection, recording and classification included in models can potentially lead to the wrong features being used for predictions and a potential bias. There is often no inherent benefit to ML models as a tool, especially when the number of predictors in a setting is low.

In addition, there is still a lack of prospective and external validation for these models. The anticipated improvement in outcomes improvement to traditional methods is marginal in some aspects of transplantation such as predicting graft pathologies after liver transplantation. Therefore, clinical integration and post-deployment monitoring of these algorithms in real-time will be necessary to evaluate the true impact of algorithms in clinical practice. In external validation studies, reductions in the predictive accuracy of models (relative to their original performance in development studies) is expected. Therefore, given the additional complexities introduced by ML algorithms, it should be ensured that models undergo rigorous but fair external validation in cohorts or simulated data. As a result, there is a potential lack of generalizability and the performance of these models should be assessed with caution before considering their application in daily clinical work, as well as more multicenter studies with distinct demographic variables, should be used as input while creating an ML model to increase it robustness on varied datasets.

For more equitable systems, studies should also consider non-clinical variables of transplantation like geographic disparity, physical compatibility between donor and recipients, and resource availability, all of which may significantly impact transplant outcomes. Depending on the ML model development, representation of the key population (by sex, age, and ethnicity) can influence the predictive accuracy of the algorithm in different subgroups, thus, creating inequities. Hence, methodologies to ensure fairness evaluation should be employed.

Another potential challenge with ML algorithms is that they are computationally complex and time-intensive, whereas some models like neural networks require more computational resources and higher number of iterations for better training. Hence, an ML model should be chosen for analysis after carefully estimating whether the ML model is better able to address key clinical questions, in a scenario where there is marginal gain in implementing a complex ML solution as compared to traditional statistical methods. Also, each ML approach has pros and cons associated with it and should be employed after careful analysis of the nature of the data and clinical questions at hand.

Another limitation of ML modeling is that they are perceived as black boxes, therefore in-depth analysis to improve the interpretability of machine-learning-based outcome predictions should be done to make ML models more clinically relevant and explainable. As supported by recent methodologies such as Local Interpretable Model-Agnostic Explanations (LIME), SHapley Additive exPlanations (SHAP), and Integrated gradients, there have been significant efforts to improve the interpretability of ML models^60–62^. How to effectively incorporate an ML algorithm in clinical care is an additional frontier to be overcome. Whereas a few key parameters can be input into biostatistical models online (online calculators including the MELD Na), ML models involve multiple clinical and laboratory values that will need to be fed into an algorithm to generate an output. Future potential of ML also lies in integrating data from various omics sources such as genetics, proteomics, and metabolomics which are high-dimensional datasets appropriate for ML to investigate nonlinear relationships as well as which have a great application in disease prediction, patient stratification, and delivery of precision medicine in the area of solid-organ transplantation.

Although ML approaches are usually evaluated in terms of their accuracy and precision which is aimed at being 100%, many factors such as the incompleteness of data sample, noise in the data and stochastic nature of the modeling algorithm can reduce the accuracy. Therefore, while application of ML approaches, the aim should be to achieve the maximum potential for the prediction ability using the dataset at hand and also tune the parameters adequately and compare to other baseline approaches to establish generalizability and robustness.

## Future directions

Training ML algorithms using multicenter data and with larger databases can decrease the risk of overfitting and provide the opportunity to examine the generalizability of these models. Therefore, larger international databases for organ transplant populations will be essential for future research in this field. Also, applying ML algorithms to molecular, genetic, and radiological data as well as their combination could inform a more personalized approach to patient management.

ML techniques hold tremendous potential to further improve the outcomes of transplant recipients, given the complexity and diversity of factors that impact health in transplant medicine. With the increasing amount of data available, and the ability of ML algorithms to uncover hidden interrelationships, these tools hold great promise in informing a precision medicine approach to transplant and improving overall outcomes.

### Summarized recommendations

- The performance of ML algorithms significantly correlates with the quality of the input data. Therefore, organizing data registries with low missing rates is imperative to the development of robust ML algorithms. Accordingly, large clinical datasets might lack the accuracy and granularity needed for ML algorithms to uncover hidden nonlinear associations.
- Training ML and DL algorithms usually require systems with high computing power which may not be accessible by hospitals. Therefore, developing models which require lower computing power and time could result in more universal application of these models in clinical settings.
- Due to heterogeneity and variation in the variables collected by different transplant data registries, it is important to fine-tune ML algorithms using new data available at every transplant center to optimize the model’s performance.
- By using ML algorithms such as Natural Language Processing (NLP), clinicians can take advantage of non-structured data (such as free texts from patient medical records and progress notes) along with structured organized data to improve organ allocation and post-transplant outcome.
- Routinely used ML algorithms may not be able to work efficiently with imbalanced datasets. Therefore, applying more recent methods such as weighted LSTM or ordinal oversampling techniques can improve the performance of ML models trained on imbalanced datasets which are very common in medical research.
- Advantages of Bayesian Additive Regression Trees (BART) compared to logistic regression models include easier pre-processing and visualization of data, as well as capturing nonlinear relationships during prediction, whereas disadvantages include high model complexity and instability in the tree in case of small changes in data.
- ANNs require large data to be trained on to estimate their predictive parameters accurately. On the other hand, the conventional logistic regression method benefits from a small number of hyperparameters in its modeling but is not suitable to capture nonlinear and complex relationships among them.
- Training ML models using identified important predictive features can improve the performance of these algorithms compared to developing models using all the available variables
- Although ML models especially ANNs used to be considered as “black box”, recent methods including Local Interpretable Model-Agnostic Explanations (LIME), SHapley Additive exPlanations (SHAP), and Integrated gradients have improved the interpretability of these algorithms by providing a list of the most important predictive variables for ultimate outcomes, enabling clinicians to assess the performance of ML algorithms based on the clinical perspective.
- Compared to other ML methods which are usually perceived as a black box, decision trees are more interpretable providing a tree-like structure distinguishing the key features from the feature set used to split the tree at each node; this model requires basic clinical variables for input to produce an algorithm guiding clinicians with their patient management.
- The CART and LASSO regression models can be used for building risk scores based on the provided coefficients, leading to the easier implementation of this algorithm in clinical practice.

### Reporting summary

Further information on research design is available in the Nature Research Reporting Summary linked to this article.

## Supplementary information

Reporting Summary

## Data Availability

Data sharing is not applicable to this article as no new data were created or analyzed in this study.
